# Combined endoscopic endonasal and sublabial transmaxillary approach for resection of giant infratemporal fossa schwannoma with intracranial extension: operative video and technical nuances

**DOI:** 10.3171/2020.4.FocusVid.19964

**Published:** 2020-04-01

**Authors:** James K. Liu, Kevin Zhao, Alejandro Vazquez, Jean Anderson Eloy

**Affiliations:** Departments of 1 Neurological Surgery and; 2 Otolaryngology–Head and Neck Surgery,; 3 Center for Skull Base and Pituitary Surgery, Neurological Institute of New Jersey, Rutgers University, New Jersey Medical School, Saint Barnabas Medical Center, RWJ Barnabas Health, Livingston and Newark, New Jersey

**Keywords:** endoscopic endonasal approach, sublabial transmaxillary, Caldwell-Luc, infratemporal fossa, schwannoma, endoscopic skull base surgery, surgical video

## Abstract

Tumors of the infratemporal fossa (ITF) are surgically formidable lesions due to their deep location and proximity to critical neurovascular structures. Selecting the optimal surgical corridor for a giant ITF lesion with extensive medial and lateral extension can be challenging due to the limited surgical freedom offered by each individual approach. In this operative video, we demonstrate a case of a 44-year-old female with a giant ITF schwannoma with intracranial extension and erosion of the central skull base. Although we considered several surgical approaches, including a standard binostril endoscopic endonasal approach and an endoscopic Denker’s approach, we eventually chose a combined endoscopic endonasal and sublabial (Caldwell-Luc) transmaxillary approach. This combined approach provides significantly greater surgical freedom than a pure endonasal route to the lateral ITF. The sublabial Caldwell-Luc corridor provides a more direct “head-on” trajectory to the target of the lateral ITF than the pure endonasal route. This combined approach provides a multiportal, multicorridor access, allowing for more surgical freedom and preservation of the piriform aperture and nasolacrimal duct. This case illustrates the versatility of the combined endoscopic endonasal and sublabial transmaxillary approach for giant ITF tumors with significant lateral extension. The technical nuances and surgical concepts are demonstrated in this operative video manuscript.

The video can be found here: https://youtu.be/gy-pkjLdDgE.

## Transcript

This is Dr. James Liu from Rutgers New Jersey Medical School. I will be demonstrating a combined endoscopic endonasal and sublabial transmaxillary approach for resection of a giant infratemporal fossa schwannoma with intracranial extension.

### 0:36 Patient history and physical examination

The patient is a 44-year-old female who presented with progressive right eye proptosis and sinonasal obstruction associated with right visual loss and facial numbness in the right V2 and V3 region. Neuro-ophthalmologic exam showed a compressive right optic neuropathy. Visual acuity was 20/20 in the left eye and only counting fingers at 2 feet in the right eye. The remainder of her neurological exam was nonfocal.

### 1:04 Preoperative imaging

CT angiogram showed a giant mass in the right infratemporal fossa and nasal cavity with erosion of the central skull base and petrous carotid canal. Right eye proptosis is seen as well. The coronal views show bone erosion of the middle fossa from intracranial extension and compression of the right optic canal and orbital floor.

MRI showed a giant brightly enhancing mass in the right infratemporal fossa with intracranial extension. The differential diagnosis included schwannoma and meningioma. An endoscopic endonasal biopsy of the mass confirmed a schwannoma.

### 1:44 Surgical approach selection

If we choose a pure binostril endoscopic endonasal approach, lateral exposure to the lateral infratemporal fossa would be quite limited. Alternatively, an endoscopic Denker’s approach can be performed to obtain more lateral exposure by removing the maxilla at the inferior lateral piriform aperture. However, we feel that this maneuver is still limited by the soft tissue of the nose and typically results in nasolacrimal duct transection. The working angle to the lateral infratemporal fossa via the nostrils is a bit awkward, and there is also a risk of a cosmetic sunken deformity of the lateral nasal alae.

Another option is to take the binostril endoscopic endonasal corridor and combine it with an anterior sublabial (Caldwell-Luc) transmaxillary corridor. This combined approach provides significantly greater surgical freedom than a pure endonasal route. It also provides a direct “head-on” trajectory to the target, which is more natural and less awkward than the pure endonasal route.

The addition of the anterior maxillotomy window now allows for multiportal, multicorridor surgery. The endoscope can be placed in the right nostril, while the working instruments can be placed in the maxillotomy and left nostril. This maximizes surgical freedom and minimizes instrument collision, or “sword fighting.” Again, the Caldwell-Luc maxillotomy provides direct anterior access to the lateral infratemporal fossa without violation of the nasolacrimal duct.

### 3:21 Patient positioning

The patient is positioned supine in pins with the head rotated to the right approximately 15°. Image guidance is used along with visual and somatosensory evoked potentials. The nose, nares, and mouth are prepped with betadine solution.

### 3:37 Maxillotomy

A right sublabial incision is made and subperiosteal elevation of the mucosa exposes the anterior maxillary wall. A Caldwell-Luc maxillotomy is performed with Kerrison rongeurs. The wall is quite thin due to pressure erosion from the large tumor. The corridor is further widened to allow maximal exposure into the transantral corridor.

### 4:01 Endoscopic endonasal exposure

The nasal mucosa is infiltrated with 1% lidocaine with 1:100,000 of epinephrine, and the right inferior turbinate is removed with a microdebrider. This endoscopic medial maxillectomy exposes the medial aspect of the tumor and communicates the nasal corridor with the transantral corridor. The middle turbinate is also removed with a microdebrider. We now start to debulk the medial aspect of the tumor using a microdebrider.

### 4:31 Resection of tumor

After debulking the tumor within the nasal corridor, we come back to the maxillotomy corridor to dissect the tumor laterally. The tumor is devascularized with a suction bovie and bipolar forceps. The superior margin of the tumor capsule is carefully peeled away from the remnant of the remodeled posterior wall of the maxillary sinus. Careful review of the sagittal CT scan shows that expansile growth of the tumor has remodeled the posterior wall of the maxillary sinus so that it has been displaced anteriorly and superiorly up against the orbital floor. Intraoperatively, there is a plane of dissection between the remodeled orbital floor and the posterior wall of the maxillary sinus. The key pearl in removing this tumor is using the posterior wall of the maxillary sinus as a plane of dissection to peel the tumor capsule off of the orbital floor. A Cottle elevator is used to define a plane between the lateral aspect of the tumor and the infratemporal fossa. After further tumor debulking, the inferior margin of the tumor is dissected away from the floor of the maxillary sinus. Once the tumor is dissected away from the posterior wall of the maxillary sinus, safe debulking can then be performed with the microdebrider. The bone is kept in place temporarily to maintain a plane of dissection around the tumor capsule.

The 30° angled endoscope can be placed in either the endonasal corridor or the transmaxillary corridor depending on the desired view of the tumor. Meanwhile, the instruments can be placed in the binostril corridor, purely in the transmaxillary corridor, or one in each corridor. This multiportal, multicorridor strategy maximizes the surgical freedom and minimizes instrument collision.

The remaining portion of tumor is adherent to the back of the infratemporal fossa. It is important to further reduce the tumor size, either by microdebriding or piecemeal scissor debulking, so that the tumor remnant is easier to manipulate and prevent catastrophic injury to surrounding neurovascular structures.

Removal of the middle fossa floor now reveals the middle fossa dura. The remaining tumor is situated between this and the infratemporal fossa. This is carefully removed and dissected away from the resection bed. Although the exact origin of the tumor is difficult to localize, it is likely that this tumor arose from a branch of V2, possibly the infraorbital nerve. The cavity is inspected and there is no evidence of residual tumor or intraoperative CSF leakage.

### 7:43 Multilayer reconstruction

Multilayered reconstruction is performed by initially placing an AlloDerm graft over the exposed skull base followed by a free mucosal graft. This is then bolstered with Surgicel, followed by gentamicin-soaked Gelfoam pledgets and a Merocel pack.

The lateral piriform aperture was fractured iatrogenically because the giant tumor had remodeled the bone. This was reconstructed with a single titanium fixation plate and screws. Closure of the sublabial incision is performed with an absorbable chromic gut suture.

### 8:37 Postoperative course and postoperative imaging

Postoperatively, the patient was essentially neurologically intact, with the exception of mild right V2 numbness, which was same as preoperative baseline. Vision improved to 20/20 on the right with resolution of right proptosis. There was no postoperative CSF leakage.

Immediate postoperative CT shows packing material in place of the removed tumor. It also confirms preservation of the true orbital floor after removing the posterior wall of the maxillary sinus.

Postoperative MRI at 6 months and 12 months showed no evidence of residual or recurrent tumor. There was no middle fossa encephalocele.

### 9:21 Conclusion

In summary, the combined endoscopic endonasal and sublabial transmaxillary approach is a versatile approach for giant infratemporal fossa tumors with lateral extension. Multiportal and multicorridor access provides significant surgical freedom while avoiding instrument collision.

## Supplemental Information

Supplemental Figs. 1 and 2

## References

[1] Couldwell WT Sabit I Weiss MH Giannotta SL Rice D Transmaxillary approach to the anterior cavernous sinus: a microanatomic study Neurosurgery 40 1307 1311 1997 9179908 10.1097/00006123-199706000-00040

[2] Eloy JA Murray KP Friedel ME Tessema B Liu JK Graduated endoscopic multiangle approach for access to the infratemporal fossa: a cadaveric study with clinical correlates Otolaryngol Head Neck Surg 147 369 378 2012 22470157 10.1177/0194599812442612

[3] Little AS Nakaji P Milligan J Endoscopic endonasal transmaxillary approach and endoscopic sublabial transmaxillary approach: surgical decision-making and implications of the nasolacrimal duct World Neurosurg 80 583 590 2013 22381853 10.1016/j.wneu.2012.01.059

[4] Liu JK Husain O Kanumuri V Khan MN Mendelson ZS Eloy JA Endoscopic graduated multiangle, multicorridor resection of juvenile nasopharyngeal angiofibroma: an individualized, tailored, multicorridor skull base approach J Neurosurg 124 1328 1338 2016 26566205 10.3171/2014.12.JNS141696

[5] Sabit I Schaefer SD Couldwell WT Extradural extranasal combined transmaxillary transsphenoidal approach to the cavernous sinus: a minimally invasive microsurgical model Laryngoscope 110 286 291 2000 10680931 10.1097/00005537-200002010-00019

[6] Theodosopoulos PV Guthikonda B Brescia A Keller JT Zimmer LA Endoscopic approach to the infratemporal fossa: anatomic study Neurosurgery 66 196 202 2010 20023550 10.1227/01.NEU.0000359224.75185.43

[7] Wilson DA Williamson RW Preul MC Little AS Comparative analysis of surgical freedom and angle of attack of two minimal-access endoscopic transmaxillary approaches to the anterolateral skull base World Neurosurg 82 e487 e493 2014 23395852 10.1016/j.wneu.2013.02.003

