# Supplementary material for: Combined endoscopic endonasal and sublabial transmaxillary approach for resection of giant infratemporal fossa schwannoma with intracranial extension: operative video and technical nuances
Source: Neurosurg Focus Video. 2020 Apr 1;2(2):V16. doi: 10.3171/2020.4.FocusVid.19964 (PMC9542375; doi:10.3171/2020.4.FocusVid.19964)
Supplement: Supplemental Figs. 1 and 2 [file 19964.Liu.NSVapr2020.supplmat.pdf]

Supplemental material

Combined endoscopic endonasal and sublabial transmaxillary approach for resection of giant infratemporal fossa schwannoma with intracranial extension: operative video and technical nuances

James K. Liu, MD,[1–3] Kevin Zhao, DO,[1] Alejandro Vazquez, MD,[2] and Jean Anderson Eloy, MD[1–3]

http://thejns.org/doi/abs/10.3171/2020.4.FocusVid.19964

DISCLAIMER *Neurosurgical Focus: Video* acknowledges that the following section is published verbatim as submitted by the authors and did not go through either the journal's peer-review or editing process.

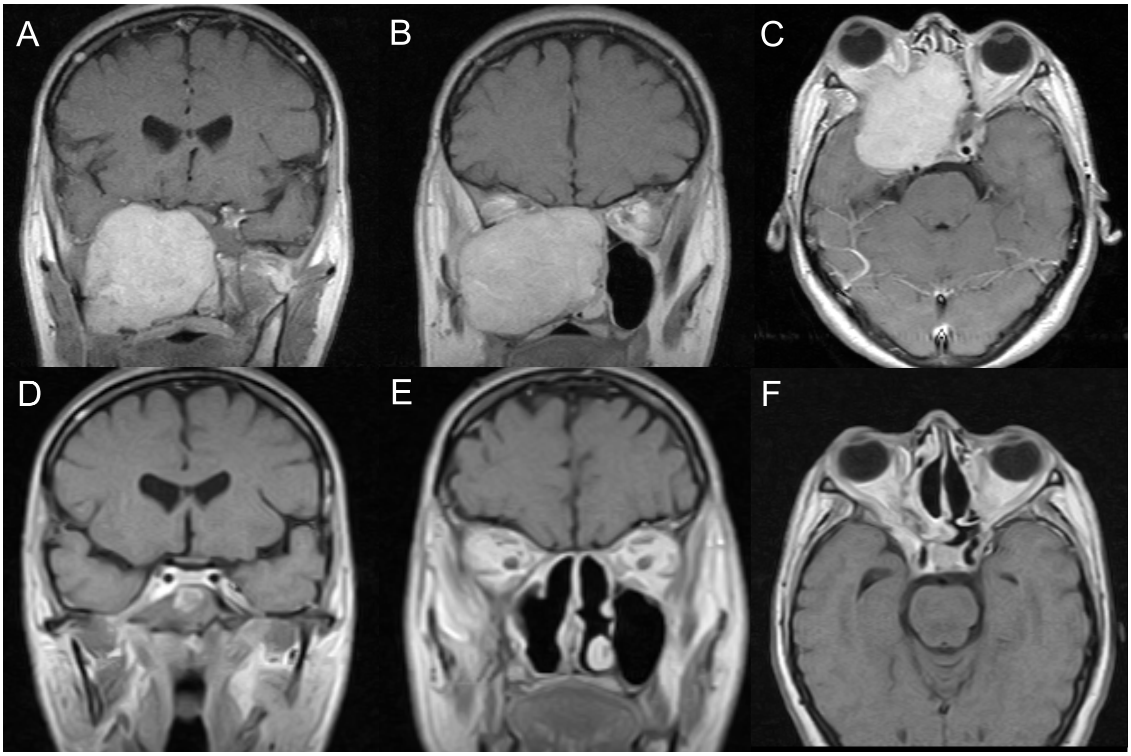

FIG. 1. Preoperative postgadolinium T1-weighted MRI coronal (A and B) and axial (C) views demonstrating a giant right infratemporal fossa schwannoma with intracranial extension. A combined endoscopic endonasal and sublabial trans-maxillary approach was performed and a gross-total resection was achieved. Postoperative postgadolinium T1-weighted MRI coronal (D and E) and axial (F) views at 6 months after surgery showing complete resection without evidence of residual tumor or recurrence.

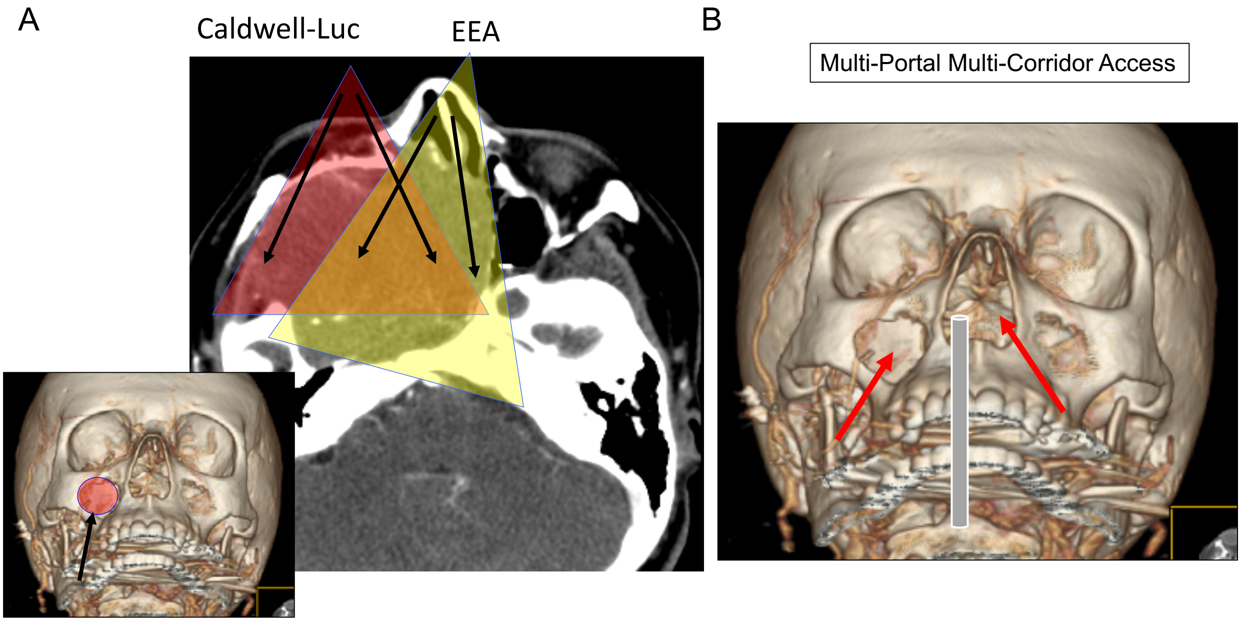

FIG. 2. A: Schematic diagram showing the operative corridors of the endoscopic endonasal and Caldwell-Luc corridors on an enhanced axial CT scan of the giant infratemporal fossa tumor. B: Three-dimensional CT of the skull shows the multiportal, multicorridor concept using the sublabial transmaxillary and binostril endonasal corridors.
